# Patient Outcomes in Crisis Resolution and Home Treatment Service: A Retrospective, Observational Study in Wolverhampton

**DOI:** 10.1192/bjo.2024.494

**Published:** 2024-08-01

**Authors:** Anurag Misra, Usman Raja, Nilamadhab Kar

**Affiliations:** 1 Penn Hospital, Wolverhampton, United Kingdom; 2 New Cross Hospital, Wolverhampton, United Kingdom

## Abstract

**Aims:**

Crisis Resolution Home Treatment Team (CRHT) provides short-term treatment for psychiatric patients in the place of residence, dealing with mental health crises, risks, deterioration, and preventing hospital admissions. This team works round the clock, seven days a week. This study aimed to analyze the clinical outcomes of the service provided by the CRHT in Wolverhampton.

**Methods:**

In a retrospective, observational, explorative study design, data was collected from the electronic medical records of 100 (54 female and 46 male) consecutive patients who were treated under the general adult (age range 18–65 years) CRHT from 1st December 2022. We collected outcome variables such as symptomatic improvement, change in risk status, days of treatment under CRHT, and discharge destination.

**Results:**

In the sample, 76% had one psychiatric diagnosis, and co-morbidities were present in 20%, with 4% of patients having no syndromal diagnoses. The most common primary diagnosis was personality disorder (24%), followed by psychotic disorders (22%), anxiety disorders 21%, and depression (20%). Overall risk status of red changed from 87% at admission to 17%, at discharge; risk to self from 43% to 17% (p < 0.01), risk to others from 11% to 1% (p < 0.01), respectively. The mean length under care of CRHT was highest with anxiety disorder (27.7 ± 18.2 days), followed by personality disorders 23.7 ± 17.9 days. Age and number of days under CRHT were negatively correlated, suggesting younger age was linked to higher number of days (p < 0.05). Most (71%) patients showed an improvement in their mental health, 15% deteriorated and got admitted to the psychiatric hospital, 9% did not engage and 5% were considered not suitable for care under CRHT for various reasons such as having no fixed abode. Most (80%) patients were discharged back to the community following the CRHT period.

**Conclusion:**

Despite the limitation of subjective clinical assessments, the results suggested that the CRHT was effective in considerable proportions of patients with symptomatic improvement and a decrease in risk level, with a small proportion being admitted to a psychiatric ward. There is a need for objective evaluation of risk and symptomatic change using validated instruments and assessing patient experiences about the services.

